# Impact of micro- and macronutrient status on the incidence of tuberculosis: An examination of an African cohort initiating antiretroviral therapy

**DOI:** 10.1371/journal.pgph.0002007

**Published:** 2023-07-13

**Authors:** Alvaro Schwalb, Malin Bergstrom, Susannah Woodd, Andrea M. Rehman, George PrayGod, Lackson Kasonka, John R. Koethe, Suzanne Filteau, Rein M. G. J. Houben

**Affiliations:** 1 Faculty of Epidemiology & Population Health, London School of Hygiene & Tropical Medicine, London, United Kingdom; 2 TB Modelling Group, TB Centre, London School of Hygiene & Tropical Medicine, London, United Kingdom; 3 Instituto de Medicina Tropical Alexander von Humboldt, Universidad Peruana Cayetano Heredia, Lima, Peru; 4 Mwanza Research Centre, National Institute for Medical Research, Mwanza, Tanzania; 5 University Teaching Hospital, Lusaka, Zambia; 6 Vanderbilt Institute of Global Health, Vanderbilt University School of Medicine, Nashville, TN, United States of America; Boston University, UNITED STATES

## Abstract

Macronutrient and micronutrient deficiencies are associated with tuberculosis (TB) incidence. However, evidence is limited on the impact of micronutrient (vitamins and minerals) supplementation among underweight individuals. We conducted a secondary data analysis of a randomised controlled trial of lipid nutritional supplements with and without high-dose vitamin and mineral supplementation (LNS-VM vs LNS) for underweight (Body Mass Index [BMI] <18.5 kg/m^2^) adults with human immunodeficiency virus (HIV) initiating antiretroviral therapy (ART) in Tanzania and Zambia (2011–2013). Incident TB disease diagnoses were extracted from trial records. We used multivariable Cox regression to estimate hazard ratios (HR) for the impact of receiving LNS-VM on TB incidence, and the dose-response relationship between baseline BMI and TB incidence. Overall, 263 (17%) of 1506 participants developed TB disease. After adjusting for age, sex, CD4 count, haemoglobin, and C-reactive protein, receiving LNS-VM was not associated with TB incidence (aHR [95%CI] = 0.93 [0.72–1.20]; p = 0.57) compared to LNS alone. There was strong evidence for an association between lower BMI and incident TB (aHR [95%CI]: 16–16.9kg/m^2^ = 1.15 [0.82–1.62] and <16kg/m^2^ = 1.70 [1.26–2.30] compared to 17–18.5kg/m^2^; linear trend p<0.01). There was strong evidence that the rate of developing TB was lower after initiating ART (p<0.01). In conclusion, the addition of micronutrient supplementation to LNS was not associated with lower TB incidence in this underweight ART-naive population.

## Introduction

Worldwide, an estimated 10 million people fell ill with tuberculosis (TB) in 2020, of whom approximately 8% were infected with the human immunodeficiency virus (HIV) [1]. This proportion is higher in Tanzania and Zambia, countries with a high joint TB/HIV burden as classified by the World Health Organization (WHO) [2]. Infection with HIV alters TB clinical presentation, accelerates disease progression, and increases the overall lifetime risk of TB incidence [3, 4]. While the lifetime risk of developing TB disease is often quoted as approximately 10%, for individuals co-infected with HIV this may rise to 5–10% per year [5, 6]. Fortunately, initiating antiretroviral therapy (ART) is effective at preventing HIV-related TB regardless of baseline CD4 counts [7].

Undernourishment is more commonly present than HIV infection, leading the list of TB determinants with 1.9 million—nearly 20% of all TB incident cases [1, 8]. There is consistent observational data that low Body Mass Index (BMI), a screening method which is used as an indicator of overall nutritional status is associated with an increase in TB mortality [9, 10]. Furthermore, previous studies have also demonstrated that lower BMI is inversely associated with TB incidence, especially in the underweight (<18.5 kg/m^2^) population [11–14]. This is thought to occur due to the impairment of cell-mediated immunity by malnutrition (including macronutrient deficiencies, i.e., proteins, carbohydrates, and fats), although the precise biological pathways underpinning this relationship are not yet fully understood [15].

Malnutrition also extends to micronutrient (i.e., vitamins and minerals) deficiencies, of which several, including vitamins D, A, C, E, zinc, and selenium, have been linked to co-prevalent TB disease [8, 16–19]. As a result, multiple trials have examined the effect of micronutrient supplementation on TB treatment outcomes; overall, the evidence has been considered insufficient [20]. It remains unclear whether this is applicable to individuals co-infected with HIV and TB, as there is little data pertaining to this specific population. Furthermore, evidence is even more limited on whether micronutrient supplementation has any impact on reducing TB incidence, partly because few studies measure TB incidence prospectively due to the usually low rate and, therefore, expense and time required [21]. Additionally, it is difficult to accurately interpret micronutrient status in inflammatory states, which confounds the assessment of the adequate absorption of micronutrient supplements and delivery to tissues needing them [22].

As with TB, malnutrition is also strongly associated with HIV [23, 24]. In terms of prognosis, individuals with both TB disease and HIV infection are likely to be more malnourished, and these may even act synergistically to worsen outcomes [24, 25]. The Nutritional Support for Adults Starting Antiretroviral Therapy (NUSTART) trial examined the effect of nutritional supplementation with or without high-dose vitamins and minerals in underweight adults living with HIV initiating ART in Tanzania and Zambia [26]. The trial found that the micronutrient supplementation had no impact on mortality in these individuals [26]. Similarly, the Trial of Vitamins-4 (ToV4) did not find an overall effect of vitamin D_3_ supplementation on the risk of mortality or overall TB incidence in adults living with HIV [27]. Despite the negative results in HIV, wide interest remains in micronutrient supplementation in the field of TB. Given the association demonstrated between micronutrients and co-prevalent TB in observational studies, it is worth exploring data from existing trials, such as NUSTART, to inform potential decisions on funding new micronutrient supplementation trials in TB. The aim of this study was to determine whether high-dose vitamin and mineral supplementation was associated with reduced incidence of TB, and whether low BMI was associated with increased incidence of TB, in a population of underweight adults living with HIV, initiating ART in Tanzania and Zambia.

## Methods

### Study design and setting

We conducted a secondary data analysis of the NUSTART randomised clinical trial conducted from August 2011 to December 2013 (Pan-African Clinical Trials Register: PACTR201106000300631, June 1, 2011) [26]. The trial was conducted in two sites: one in Mwanza, Tanzania, and the other in Lusaka, Zambia; eligible participants attending free HIV testing services at both study sites were recruited. Participants were individually randomised to receive a lipid-based nutritional supplement either without (LNS) or with high-dose vitamin and mineral supplementation (LNS-VM). This secondary data analysis was performed in two parts. The first was an analysis of the effect of LNS-VM on the secondary outcome of TB incidence in the randomised population of the original trial. The second was a prospective cohort analysis using the trial population to determine whether baseline BMI was associated with TB incidence.

### Study population

The study population comprised non-pregnant adults aged ≥18 years with HIV originally recruited to the NUSTART trial. All were underweight (BMI <18.5 kg/m^2^), eligible for ART according to guidelines at the time of the study (CD4 count <350 cells/mm^3^ or WHO stage 3 or 4 HIV/AIDS disease) and had had no previous ART treatment apart from short-course regimens for prevention of mother-to-child transmission. All participants who reported a pre-existing TB diagnosis or who reported taking treatment for TB at the first study visit were excluded from the present analysis of incidence.

### Procedures and participant follow-up

At the screening visit, baseline sociodemographic and clinical data were collected from participants and included the measurement of BMI, calculated from the median of three repeat measurements of both height and weight. Venous blood samples also provided measurements of haemoglobin (Hb), CD4 count, and C-reactive protein (CRP). Prior to the initiation of ART, participants were counselled appropriately and screened for opportunistic infections as part of routine local medical care. From recruitment to two weeks after commencing ART, a low-calorie supplement (approximately 150 kcal/day) was provided. From two to six weeks after commencing ART, a higher calorie supplement (1,400 kcal/day) with the same vitamin and mineral content was provided. After the initiation of ART, participants were followed up at 2, 4, 6, 8, and 12 weeks. Those who did not attend were additionally followed up via phone and/or home visits. Further details on the trial and the exact content of the supplements can be found in the trial publication [26].

### Assessment of outcome

The primary outcome for this secondary analysis was incident TB disease, which was ascertained in three different ways. Firstly, participants were asked if they had a new diagnosis of TB in the preceding week or were taking TB treatment at all routine study clinic visits. Secondly, information was collected on local hospital admissions; any new diagnoses of TB that were made were noted. Thirdly, any deaths due to TB were recorded if the cause of death had been provided by hospitals but not if reported by relatives alone.

### Statistical analysis

Data were anonymised prior to analysis. BMI was calculated as weight (kilograms) divided by the square of height (metres) and categorised into three groups (17–18.5, 16–16.9, and <16 kg/m^2^) [28]. Baseline characteristics of participants per intervention arms were presented as frequencies and percentages. Cox proportional hazards regression was performed for both aims—to assess the association between the trial arms and TB disease incidence, as well as between baseline BMI category and TB disease incidence. Analysis of the randomised comparison used intention-to-treat principles. Categorical covariables identified *a priori* as potential confounders to the association between micronutrient supplementation and BMI with TB incidence (age, sex, CD4 count) were included in the regression models; additionally, variables subsequently found to be confounders, and which were not multicollinear, were also added. Although covariables were randomised across groups, these were included in the multivariable analyses to increase power. Missing covariables were handled as a complete-case analysis. Tests either for general association or for linear trend, as appropriate, were presented. The proportional hazards assumption was examined both by using cumulative hazard curves and by formally testing for interaction with follow-up time using likelihood ratio tests (LRT) in Cox models. Effect modification was tested for variables identified as of interest *a priori* (sex, CD4 count). In the case of exposure to ART, the variable was split into a binary pre-ART and post-ART as a time-dependent variable, to evaluate the follow-up of participants during these two distinct periods. Whilst power was limited, the potential interaction between exposure to ART and the effect of both the intervention arm and baseline BMI group on TB disease incidence was explored. Data curation was performed in Stata v.17.0 and analyses were conducted in R v.4.1.0.

### Sensitivity analyses

Since TB disease was not systematically excluded through chest film, sputum smear, culture, or another diagnostic tool at baseline, we performed sensitivity analyses with Cox proportional hazards regression which only included participants with outcomes reported 8 weeks after enrolment, thus excluding individuals that likely had TB at study entry.

### Ethical considerations

Ethical approval for this secondary data analysis was secured from the MSc Research Ethics Committee at the London School of Hygiene & Tropical Medicine (LSHTM)(Ref. 15738). Ethical approval for the NUSTART trial was obtained from the University of Zambia Biomedical Research Ethics Committee (009-01-11), the Medical Research Coordinating Committee of National Institute for Medical Research, Tanzania (NIMR/HQ/R.8a/Vol. IX/1102), and the Research Ethics Committee at LSHTM (Ref. 5876). Written or thumbprint informed consent was obtained from all participants [26].

## Results

### Participant characteristics and follow-up

**Fig 1:**
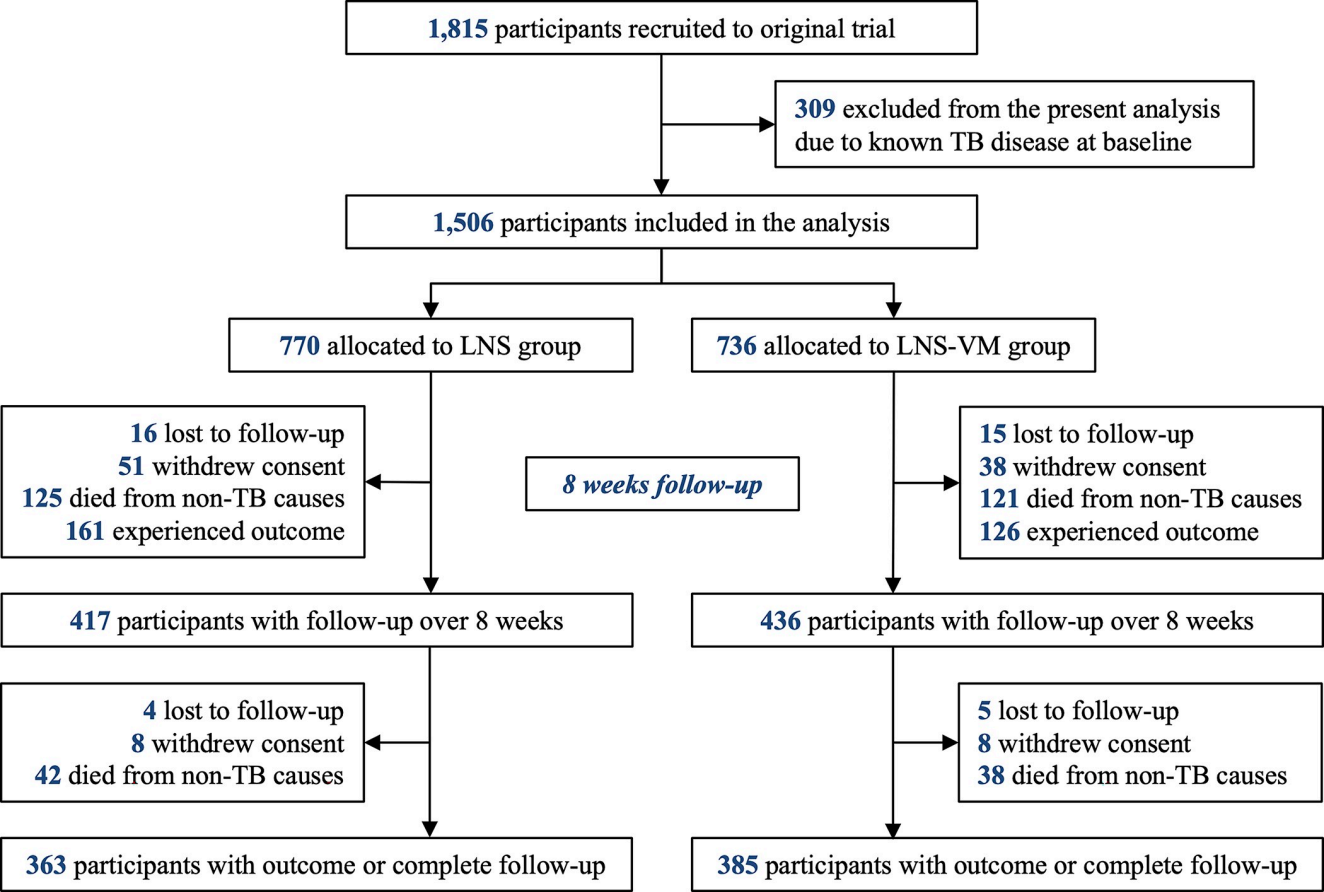
Flowchart of participants. Lipid-based nutritional supplements without (LNS) or with vitamin and mineral supplementation (LNS-VM) intervention.

The original NUSTART trial recruited 1,815 participants, of which 309 were excluded from this analysis due to known TB disease at study entry. A total of 1,506 participants were included in this secondary analysis and were followed up for 275 person-years (median follow-up time was 70 days). The follow-up time was 136 and 138 person-years for the LNS and LNS-VM group, respectively. During the study period, 105 (6.9%) participants withdrew their consent and 40 (2.6%) were lost to follow-up and so were censored **(Fig 1)**. By week eight of follow-up, 43% of participants had either experienced the outcomes or had left the study.

There was a fairly equal distribution of participants in regard to sex, and the median age was 35 years (IQR:29.8–41.5). Over half (893, 59.3%) of the participants were based in Lusaka, with the remaining being based in Mwanza (613, 40.7%). All participants were underweight as per inclusion criteria, with 490 (32.5%) participants in the BMI <16 kg/m^2^ range. There was a high prevalence of anaemia, with 71.9% having either moderate or severe anaemia. CD4 counts amongst these participants were low, with 848 (56%) individuals having a count of <100 cells/mm^3^. **Table 1** further describes the baseline characteristics of the study participants per intervention arm and according to follow-up. Participants with outcomes reported 8 weeks after enrolment had fewer individuals with BMI<16.0 kg/m^2^ (26% vs. 33%) and severe anaemia (16% vs. 22%) compared with the full cohort at baseline. For these participants between intervention arms, there was a difference in the levels of Hb and CRP.

**Table 1:** Baseline characteristics of study participants.

Characteristic		All participants		Outcome after 8 weeks		
		LNS (n = 770)	LNS-VM (n = 736)	LNS (n = 417)	LNS-VM (n = 436)	P- value*
Follow-up, person-years		136	138	116	122	-
Age group, years	18–29	198 (25.7)	183 (24.9)	100 (24.0)	103 (23.6)	0.99
	30–44	442 (57.4)	428 (58.1)	231 (55.4)	243 (55.7)	
	≥45	130 (16.9)	125 (17.0)	86 (20.6)	90 (20.6)	
Sex	Female	394 (51.2)	375 (50.9)	227 (54.4)	246 (56.4)	0.56
	Male	376 (48.8)	361 (49.1)	190 (45.6)	190 (43.6)	
Socio-economic status tertile	Low	266 (34.5)	237 (32.2)	135 (32.4)	135 (31.0)	0.31
	Middle	241 (31.3)	260 (35.3)	131 (31.4)	158 (36.2)	
	High	263 (34.2)	239 (32.5)	151 (36.2)	143 (32.8)	
Body Mass Index group, kg/m^2^	17–18.5	329 (42.7)	308 (41.9)	207 (49.6)	206 (47.2)	0.49
	16–16.9	189 (24.6)	190 (25.8)	98 (23.5)	118 (27.1)	
	<16	252 (32.7)	238 (32.3)	112 (26.9)	112 (25.7)	
Haemoglobin group^¶^, g/L	Severe anaemia (<80)	169 (23.8)	166 (24.4)	62 (16.0)	71 (17.8)	0.02
	Moderate anaemia (80–109)	326 (45.8)	339 (49.8)	171 (44.2)	206 (51.6)	
	Mild anaemia/normal (≥110)	216 (30.4)	176 (25.8)	154 (39.8)	122 (30.6)	
CD4 count group, cells/mm^3^	<100	433 (56.2)	415 (56.4)	246 (59.0)	272 (62.4)	0.31
	≥100	337 (43.8)	321 (43.6)	171 (41.0)	164 (37.6)	
C-reactive protein group^¶^, mg/L	<10	172 (23.0)	140 (19.7)	136 (34.0)	108 (25.6)	0.03
	10–160	374 (50.0)	381 (53.6)	196 (49.0)	238 (56.4)	
	>160	202 (27.0)	190 (26.7)	68 (17.0)	76 (18.0)	

Values are n (%). LNS: lipid-based nutritional supplement; LNS-VM: lipid-based nutritional supplement with high-dose vitamin and mineral supplementation.

¶ Missing data: haemoglobin group (n = 114), C-reactive protein group (n = 47).

* Chi-squared test.

Ultimately, 1,181 (78.4%) participants started ART; of the 325 for whom we have no record of this, some died or were lost to follow-up before ART initiation, and some did not want to start treatment. During follow-up, 288 (19.1%) individuals were admitted for an overnight hospital stay at least once. There were 306 (20.3%) recorded deaths, resulting in a mortality rate of 11.9 deaths per 10 person-years (95%CI:10.6–13.2). Of these, 60 (19.6%) were documented to have died of TB. There were 263 (17.5%) incident cases of TB, resulting in an overall incidence rate of 9.6 cases per 10 person-years (95%CI:8.4–10.1).

### Effect of nutritional intervention on TB incidence

**Fig 2:**
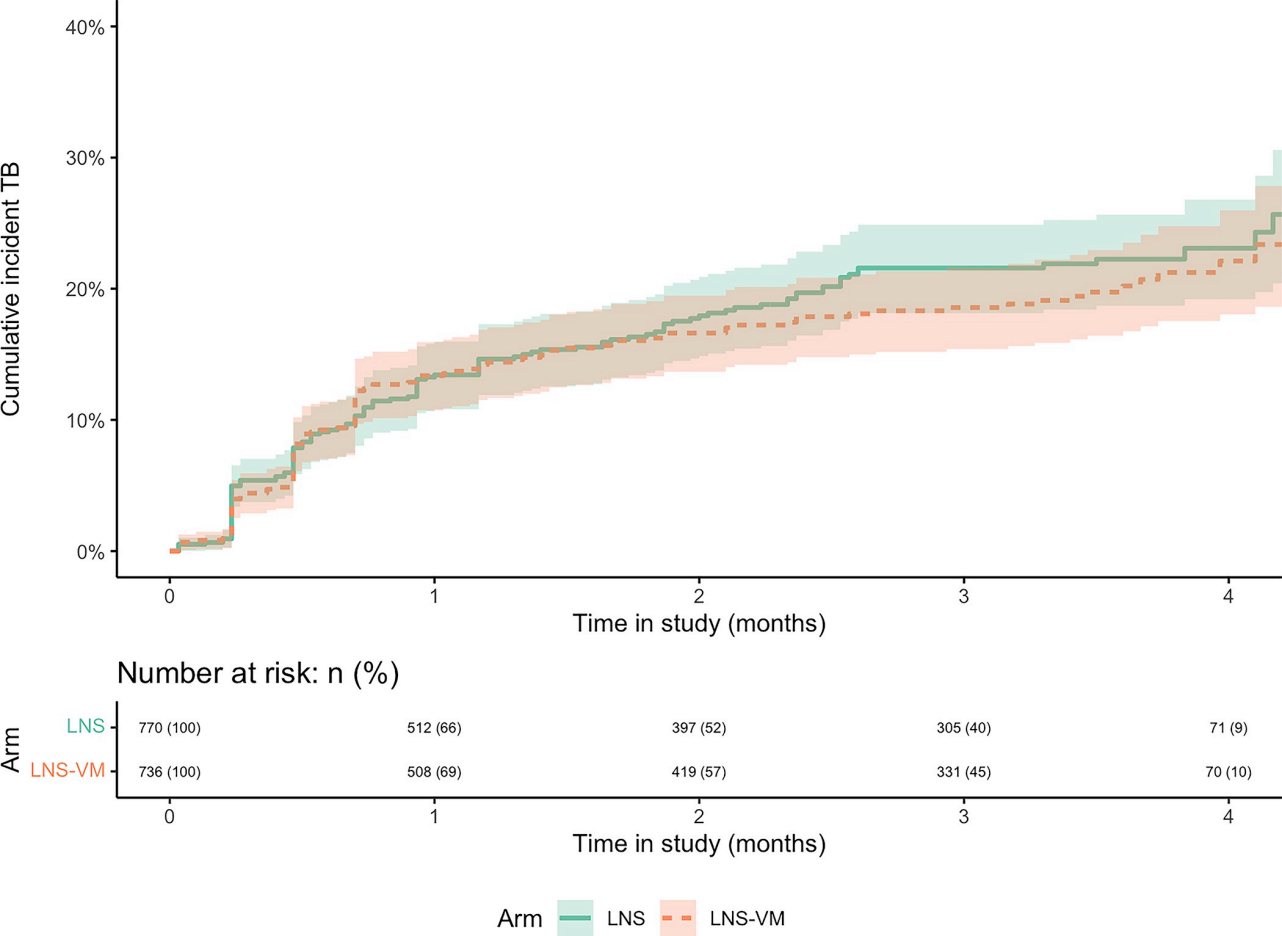
Cumulative incidence curves comparing the effect of the lipid-based nutritional supplements without (LNS) or with vitamin and mineral supplementation (LNS-VM) intervention arms on the incidence of tuberculosis disease. Logrank test p = 0.55. Time in the study displayed limited to 4 months (from 6 months) due to subsequent data sparsity.

There was no evidence for a difference in the incidence of TB disease between the two intervention arms, with rates of 10.1 cases per 10 person-years (95%CI:8.4–11.9) in the LNS group and 9.1 cases per 10 person-years (95%CI:7.6–10.9) in the LNS-VM group (cHR 0.93; 95%CI:0.73–1.18; p = 0.55) **(Fig 2)**. Similarly, after adjusting for age, sex, BMI group, CD4 count, Hb, and CRP there was no evidence for an association between the intervention arms and TB incidence (aHR 0.93; 95%CI:0.72–1.20; p = 0.57) **(Table 2)**. In the sensitivity analysis, after 8 weeks since enrolment and for similar adjustments, there was some evidence of a protective effect of LNS-VM on TB incidence compared to LNS (aHR 0.51; 95%CI:0.27–0.94) **(Table 3)**.

**Table 2:** The effect of the vitamin and mineral supplementation in lipid-based nutritional supplements on the incidence of tuberculosis disease.

Arm	Incident TB cases	Rate^†^ (95%CI)	cHR (95%CI)	P-value^‡^	aHR^¶^ (95%CI)	P-value^‡^
LNS	137	10.1 (8.4–11.9)	Ref.	0.55	Ref.	0.57
LNS-VM	126	9.1 (7.6–10.9)	0.93 (0.73–1.18)		0.93 (0.72–1.20)	

LNS: lipid-based nutritional supplement; LNS-VM: lipid-based nutritional supplement with high-dose vitamin and mineral supplementation; TB: tuberculosis; CI: confidence interval; cHR: crude hazard ratio; aHR: adjusted hazard ratio.

† Rate per 10 person-years.

‡ Obtained from likelihood test for general association.

¶ Adjusted for age group, sex, Body Mass Index group, CD4 count group, haemoglobin group, and C-reactive protein group.

**Table 3:** The effect of the vitamin and mineral supplementation in lipid-based nutritional supplements on the incidence of tuberculosis disease after 8 weeks since baseline.

Arm	Incident TB cases	Rate^†^ (95%CI)	cHR (95%CI)	P-value^‡^	aHR^¶^ (95%CI)	P-value^‡^
LNS	28	2.4 (1.6–3.5)	Ref.	0.28	Ref.	0.03
LNS-VM	21	1.7 (1.1–2.6)	0.73 (0.41–1.29)		0.51 (0.27–0.94)	

LNS: lipid-based nutritional supplement; LNS-VM: lipid-based nutritional supplement with high-dose vitamin and mineral supplementation; TB: tuberculosis; CI: confidence interval; cHR: crude hazard ratio; aHR: adjusted hazard ratio.

† Rate per 10 person-years.

‡ Obtained from likelihood test for general association.

¶ Adjusted for age group, sex, Body Mass Index group, CD4 count group, haemoglobin group, and C-reactive protein group.

### BMI association with TB incidence

**Fig 3:**
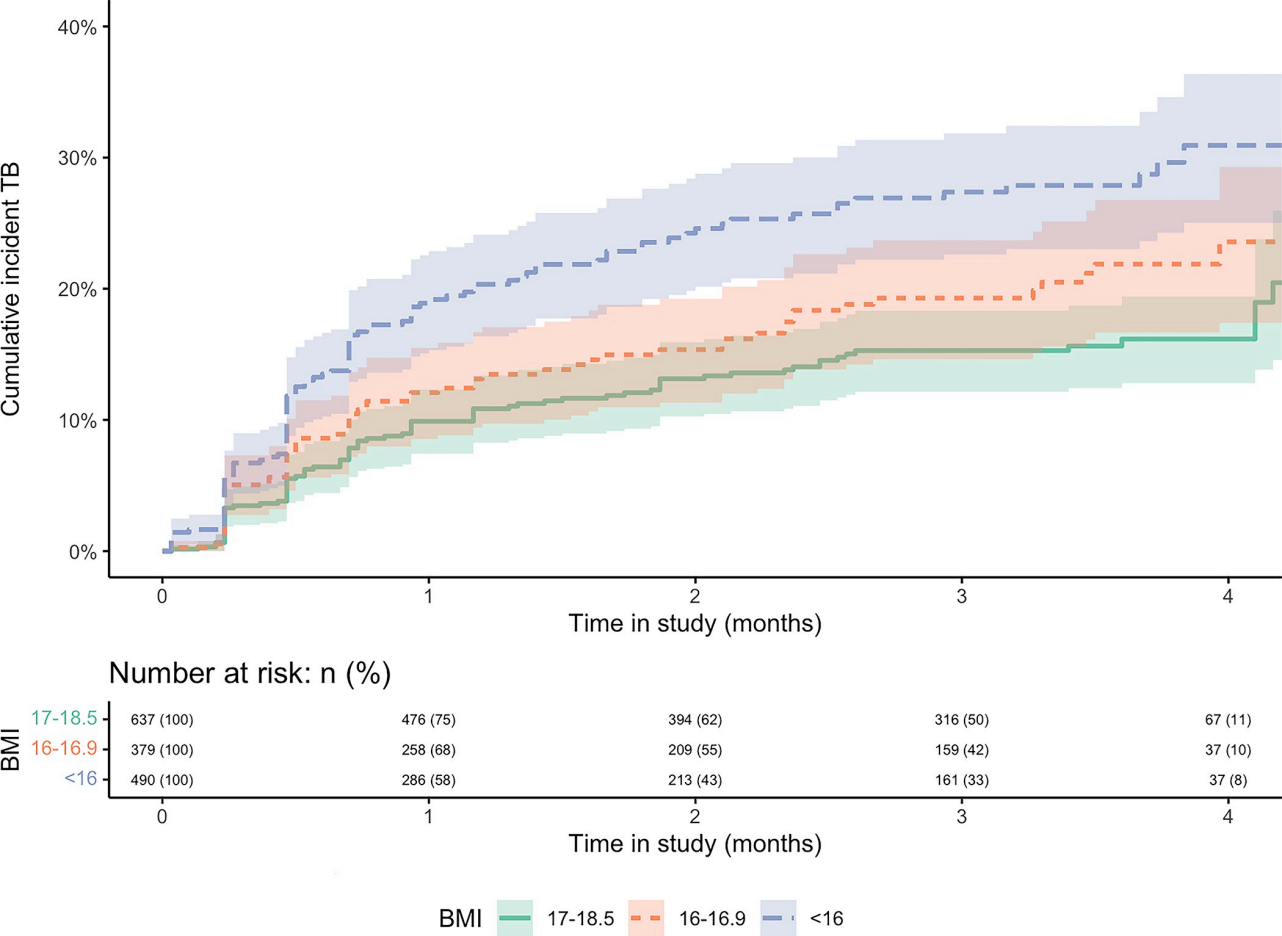
Cumulative incidence curves comparing the effect of baseline Body Mass Index (BMI, kg/m^2^) categories on the incidence of tuberculosis disease. Logrank test p<0.01. Time in the study displayed limited to 4 months (from 6 months) due to subsequent data sparsity.

After adjusting for age, sex, CD4 count, Hb and CRP, there was strong evidence that lower baseline BMI levels were associated with an increased incidence of TB disease (LRT for general association p<0.01); additionally, there was evidence of a linear trend (logrank test for linear trend p<0.01) **(Fig 3)**. Compared to the group with a BMI of 17–18.5 kg/m^2^ at recruitment, those in the 16–16.9 kg/m^2^ BMI group had a 15% increased rate of incident TB disease, whilst those in the <16 kg/m^2^ BMI group had a 70% increased rate (aHR 1.15; 95%CI:0.82–1.62; and aHR 1.70; 95%CI:1.26–2.30, respectively) **(Table 4)**. In the sensitivity analysis, after 8 weeks since enrolment and for similar adjustments, there was no evidence of an association of baseline BMI with TB incidence **(Table 5)**. In *a priori* stratified regression analyses, there was no evidence that sex or CD4 count modified the effect of BMI on TB (LRT for interaction p = 0.60 and 0.44, respectively).

**Table 4:** The effect of baseline Body Mass Index on the incidence of tuberculosis disease.

BMI group	Incident TB cases	Rate^†^ (95%CI)	cHR (95%CI)	P-value^‡^	aHR^¶^ (95%CI)	P-value^‡^
17–18.5	89	6.9 (5.5–8.5)	Ref.	<0.01	Ref.	<0.01
16–16.9	64	9.3 (7.1–11.8)	1.30 (0.95–1.80)		1.15 (0.82–1.62)	
<16	110	14.5 (11.9–17.4)	1.93 (1.46–2.55)		1.70 (1.26–2.30)	

BMI: Body Mass Index (kg/m^**2**^); TB: tuberculosis; CI: confidence interval; cHR: crude hazard ratio; aHR: adjusted hazard ratio.

† Rate per 10 person-years.

‡ Obtained from likelihood test for general association.

¶ Adjusted for age group, sex, CD4 count group, haemoglobin group, and C-reactive protein group.

**Table 5:** The effect of baseline Body Mass Index on the incidence of tuberculosis disease after 8 weeks since baseline.

BMI group	Incident TB cases	Rate^†^ (95%CI)	cHR (95%CI)	P-value^‡^	aHR^¶^ (95%CI)	P-value^‡^
17–18.5	20	1.7 (1.0–2.6)	Ref.	0.41	Ref.	0.52
16–16.9	15	2.5 (1.4–4.1)	1.52 (0.77–2.99)		1.48 (0.71–3.11)	
<16	14	2.3 (1.2–3.8)	1.39 (0.69–2.78)		1.37 (0.66–2.83)	

BMI: Body Mass Index (kg/m^**2**^); TB: tuberculosis; CI: confidence interval; cHR: crude hazard ratio; aHR: adjusted hazard ratio.

† Rate per 10 person-years.

‡ Obtained from likelihood test for general association.

¶ Adjusted for age group, sex, CD4 count group, haemoglobin group, and C-reactive protein group.

### Examining ART initiation as a time-dependent variable

There was strong evidence that the rate of developing TB varied before and after initiating ART (LR chi-square test p<0.01). After participants commenced ART, the rate was approximately 70% lower than before they started ART (HR 0.29; 95%CI:0.20–0.43). In addition, there was weak evidence that the effect of the LNS-VM on TB disease incidence varied in the periods pre- and post-ART initiation (LRT test for interaction p = 0.06). Prior to initiating ART, there was no difference in TB incidence among participants receiving the LNS-VM compared to those receiving LNS (HR 1.00; 95%CI:0.75–1.35). Similarly, after initiating ART, there was no difference in TB incidence among those receiving LNS-VM compared to LNS (HR 0.76; 95%CI:0.49–1.18). Additionally, there was no evidence that these distinct periods modified the effect of BMI on TB disease incidence (LRT for interaction p = 0.72).

## Discussion

In this secondary analysis of the data from the NUSTART trial, high-dose vitamin and mineral supplementation was not associated with lower TB incidence among underweight adults eligible to start ART in Tanzania and Zambia. However, in stratified analyses testing for interaction with ART exposure, there was some suggestion that it led to reduced TB disease incidence after participants commenced ART. This may indicate that ART-associated immune recovery could enable this protective effect, but analyses were underpowered. Contrastingly, this study finds a strong dose-response association with TB incidence across the low BMI categories which parallels that of the existing literature [13]. Moreover, in this cohort, there was a high mortality rate of 11.9 deaths per 10 person-years and an extremely high TB incident rate of 9.6 cases per 10 person-years, which is higher than both national averages [29].

Micronutrient supplementation for TB incidence among an HIV-positive population has been explored previously. One study suggested that selenium supplementation, with or without other multivitamins, reduced the incidence of TB among an ART-naive cohort; multivitamins alone did not affect incidence [30]. However, given that participants in that trial were ineligible for ART since they had non-advanced HIV infection at the time, one could argue that LNS-VM did not perform well for TB incidence as the NUSTART population was generally more unwell. It is also worth noting that the period immediately pre- and post-ART initiation is a high-risk period for TB incidence, which is expected to decrease with time [31].

The strengths of this analysis include the large sample size for a study of this kind (even after excluding pre-existing TB), the randomisation of the intervention, and its prospective nature of investigating TB incidence as an outcome. In general, TB incidence studies are lengthy and expensive, and it is resource-intensive to conduct a dedicated cohort study or trial to answer such questions. In contrast, this analysis has made use of prospective data available from a previous trial and has therefore been able to take advantage of its large numbers in an efficient manner.

Nonetheless, secondary data analysis also carries limitations. Considering the natural history of TB and HIV, it is likely that both infections were present before enrolment, particularly among individuals with low CD4 counts; this severe immunosuppression could lead to a flare up of existing *Mycobacterium tuberculosis* infection. Whether prevalent TB disease was appropriately excluded at the start of the trial was of particular concern as diagnostic tests for TB were only carried out as part of standard clinical practice, not as trial procedures, as the original trial was mainly focused on HIV and nutrition. The main assumption made in this study was that any cases detected after excluding co-prevalent cases during recruitment were incident cases of TB. A short average follow-up time coupled with the long incubation period of TB infection does raise the potential of misclassification of the outcome. However, two important factors appear to mitigate this possibility. Firstly, TB displays an accelerated natural history in individuals who are co-infected with HIV, with the progression from *Mycobacterium tuberculosis* infection to TB disease occurring over weeks to months instead of years [4]. Additionally, not only speed but overall lifetime risk of TB disease is increased when infected with HIV [6]. For the BMI analysis, there was no evidence that ART initiation (i.e., being in the pre/ post-ART phases of the trial) modified the effect of BMI on TB incidence. This is meaningful as it shows that baseline BMI is a substantial risk factor for incident TB regardless of the effect of the period immediately after ART initiation.

Our sensitivity analyses excluding participants diagnosed with TB within the first eight weeks after enrolment differ greatly from those including all participants. Unlike the primary analysis, there was some evidence of a protective effect of LNS-VM while there was no longer any evidence of an association between baseline BMI and TB incidence. This is likely because the most ill and undernourished individuals died within the first weeks and would represent a different population than that after 8 weeks. This is supported by the fact that those still in the study at 8 weeks had lower proportions with severe anaemia or BMI<16.0 kg/m2 compared with the full cohort at baseline. It should be highlighted that the 8-week cut-off was arbitrarily chosen to sit in (roughly) the middle of the total time for participants completing the follow-up (~15 weeks). Notwithstanding, given the large drop in sample size (43%) and potential unmeasured differences between populations, the sensitivity results should be interpreted carefully.

Similarly, a second limitation was the potential for the differential measurement error of TB disease during the trial. While the study attempts to remedy this by using three methods to assess the outcome, these were not as robust as if they had been done as study procedures and thus ensuring that assessment did not differ between groups. In the BMI analysis, the possibility that clinicians would be more likely to examine very underweight individuals for TB and send them for further investigation would lead to an overestimation of the effect size observed. This seems unavoidable as weight loss is a recognised symptom of TB and rightly forms part of the diagnostic process. However, again, the fact that the association of baseline BMI with TB incidence was not modified by starting ART goes against this, as one would then have expected the effect to wane after initiating ART. Of note, the main analysis on micronutrient supplementation in the randomised population is not expected to have been affected by such measurement error. On the other hand, it can be argued that there needs to be longer follow-up to see a significant effect of micronutrient supplementation on TB incidence. For example, in the case for ART, protection is fully established after 2 years since initiation [32]. This should motivate the design of future nutrition clinical trials featuring longer follow-ups to fully capture participant outcomes.

Furthermore, compliance to the nutritional supplements was only modest; the original NUSTART trial reported that less than half of participants consumed at least 75% of the expected number of the supplement sachets during their time in the study [26]. In a sensitivity analysis restricted to participants with high adherence, there were differences in mortality rates; however, there was no evidence of an effect of micronutrient supplementation. Given that the outcome in our secondary analysis occurred less frequently than the reported deaths, we do not expect that we would see a significant effect of micronutrient supplementation with the risk of TB incidence in this subpopulation.

Finally, in terms of generalisability, it is important to note that this study population comprised an underweight cohort of individuals living with HIV with very low CD4 counts. Fortunately, this population is in decline due to wider access to ART since the move towards the universal treatment of HIV in 2015 [33]. Conclusions drawn from this study may therefore not apply to other populations, but similarly do provide a note of caution for the potential of micronutrient supplementation for reducing TB incidence in low-income settings. Our findings also contribute to the growing epidemiological knowledge base surrounding the link between nutrition and TB.

In conclusion, in this population of underweight individuals living with HIV, TB incidence was high and vitamin and mineral supplementation did not reduce the overall incidence of TB disease, although there was some evidence to suggest that immune recovery in the form of ART could enable a protective effect of micronutrients. Furthermore, TB incidence increased with decreasing BMI. On the other hand, the results from the sensitivity analyses indicate the opposite. Our results provide a reason to be cautious of expecting observational results on the benefits of micronutrient supplementation to persist in an interventional study. Given that more than one in five cases of TB are attributable to undernutrition, it is sensible to expect that future studies will continue to investigate the effect of nutritional supplementation on TB-related outcomes [1]. Addressing overall undernutrition, i.e., low BMI, remains likely effective [34]. Food insecurity is common in TB households globally and has also been shown to predict who will develop TB among household contacts [35]. Addressing these wider socioeconomic determinants of TB may be a more efficient use of resources in research.
